# The effect of referral templates on out-patient quality of care in a hospital setting: a cluster randomized controlled trial

**DOI:** 10.1186/s12913-017-2127-1

**Published:** 2017-03-07

**Authors:** Henrik Wåhlberg, Per Christian Valle, Siri Malm, Øistein Hovde, Ann Ragnhild Broderstad

**Affiliations:** 1 Department of Community Medicine, UiT The Arctic University of Norway, 9037 Tromsø, Norway; 2 University Hospital of North Norway Harstad, St. Olavsgate 70, 9480 Harstad, Norway; 3 Department of Clinical Medicine, UiT The Arctic University of Norway, 9037 Tromsø, Norway; 4 Department of Gastroenterology, Innlandet Hospital Trust, 2819 Gjøvik, Norway; 5 Institute for Clinical Medicine, University of Oslo, P.O. Box 1171, 0318 Oslo, Norway; 6 Centre for Sami Health Research, UiT The Arctic University of Norway, 9037 Tromsø, Norway

**Keywords:** Quality of care, Referral, Care cooperation

## Abstract

**Background:**

The assessment of quality of care is an integral part of modern medicine. The referral represents the handing over of care from the general practitioner to the specialist. This study aimed to assess whether an improved referral could lead to improved quality of care.

**Methods:**

A cluster randomized trial with the general practitioner surgery as the clustering unit was performed. Fourteen surgeries in the area surrounding the University Hospital of North Norway Harstad were randomized stratified by town versus countryside location. The intervention consisted of implementing referral templates for new referrals in four clinical areas: dyspepsia; suspected colorectal cancer; chest pain; and confirmed or suspected chronic obstructive pulmonary disease. The control group followed standard referral practice. Quality of treatment pathway as assessed by newly developed quality indicators was used as main outcome. Secondary outcomes included subjective quality assessment, positive predictive value of referral and adequacy of prioritization. Assessment of outcomes was done at the individual level. The patients, hospital doctors and outcome assessors were blinded to the intervention status.

**Results:**

A total of 500 patients were included, with 281 in the intervention and 219 in the control arm. From the multilevel regression model the effect of the intervention on the quality indicator score was insignificant at 1.80% (95% CI, −1.46 to 5.06, *p* = 0.280). No significant differences between the intervention and the control groups were seen in the secondary outcomes. Active use of the referral intervention was low, estimated at approximately 50%. There was also wide variation in outcome scoring between the different assessors.

**Conclusions:**

In this study no measurable effect on quality of care or prioritization was revealed after implementation of referral templates at the general practitioner/hospital interface. The results were hindered by a limited uptake of the intervention at GP surgeries and inconsistencies in outcome assessment.

**Trial registration:**

The study was registered under registration number NCT01470963 on September 5th, 2011.

**Electronic supplementary material:**

The online version of this article (doi:10.1186/s12913-017-2127-1) contains supplementary material, which is available to authorized users.

## Background

Quality of care is now an integral part of modern medicine, exemplified most recently in Norway by the National Patient Safety Programme [1], a national strategy for quality improvement in health and social services [2], and several national registries [3, 4]. To define quality in health care, though, is challenging because of its subjective nature [5]. The definition by Donabedian is “the application of medical science and technology in a manner that maximises its benefit to health without correspondingly increasing the risk” [6]; in many ways this represents what many physicians regard as high-quality care. Others have highlighted the need to take patient expectations and financial constraints into account in the definition of quality of care [7].

Measurement of quality is important both to ensure the quality of services and to aid hospital management. Several authors have highlighted the usefulness of quality measurement in documenting the quality of care, making comparisons, prioritizing, quality improvement, and accountability [8, 9]. However, as indicated in a recent editorial, care must be taken to ensure that quality—not the measuring of quality—remains the aim [10]. Quality measures are usually classified as structural, process, or outcome measures [11]. Structural measures are often easy to evaluate, and examples include equipment, facility, and staffing numbers. However, they tend to be weakly associated with outcomes [12]. Process measures are the components of the encounter between the patient and health-care professional, such as ordered tests [9, 13]. They aim to assess how often an intervention known to correlate with a favourable outcome takes place. Outcome measures use the health outcome, such as survival, complications, and quality of life, as a quality indicator [12]. The use of outcome measures is impeded by several factors, such as the infrequent occurrence of some events (e.g. mortality and morbidity), and the fact that the interval between intervention and event may extend for years [12, 14].

Many quality criteria have been developed [15–18], often following the RAND Corporation/UCLA (University of California) appropriateness method [19], but other methods have also been described [20, 21]. A perfect quality measure would fully correlate with positive outcomes for each individual patient. However, an error-free measure of quality of care is unlikely ever to be created [9]. Quality indicators are only “tools that can support quality improvement”—not necessarily direct measures of quality [22]. Some authors have expressed concern about quality measures that focus on small aspects of care; they fear that other aspects of equal, or greater, importance may receive less attention [23, 24]. In a recent article, Bishop emphasized that quality measurement in outpatient care is incomplete and that the focus is mainly on preventive care, chronic disease management, and patient experience [25]. Similarly, a review of performance measures in the specialist referral process identified multiple measures [26]; most of these concentrated, though, on the structural components of the referral process—as opposed to holistically depicting the quality of the entire treatment process.

The referral constitutes the handing over of care from one caregiver to another. For the purpose of this paper referral is defined as the handing over of care from the general practitioner (GP) to secondary care. To assure high quality further down in the treatment process, the referral letter from the GP should contain all the necessary information in a context of shared understanding among the GP, the patient, and hospital staff [27]. However, a number of publications have pointed to the varying quality and content of referrals in clinical practice [28–30]. Over the years, many interventions have been directed at the referral process. A Cochrane review on this subject indicates the complexities of research in this field and states that surprisingly few interventions on the referral system have been rigorously evaluated [31].

In Norway, the health-care system is relatively uniform throughout the country. Each GP has a list of patients for whom he/she provides care. GPs act as gatekeepers to secondary care [32]. Specialist health care is delivered through governmentally owned regional health authorities—mainly via public hospitals, but private health care is available to some extent. Communication between GPs and hospitals is almost exclusively electronic, with the automatic retrieval of demographic information, such as addresses, contact details, and GP details in each referral, according to a national standard [33]. Apart from this automatic retrieval referrals are, in normal practice, mainly written in free text format containing the information each referring GP deem necessary. Beyond basic demographical information the referrals therefore contain varying amount and type of clinical information.

According to several of the aspects indicated in the Cochrane report mentioned above [31], the current study is an attempt to evaluate a referral intervention in a setting with a well-organized GP care system electronically linked to the local hospital. Those aspects include referral quality, secondary-care management of patients, and patient outcomes and satisfaction. We have previously shown that the quality of the referrals in the intervention group improved by 18% (95% CI 11, 25), *p* < 0.001 [34]. This increase however, is of limited value unless it translates into a measurable change in outcomes that matter to patients and caregivers. The current article presents the effect of this increase in referral quality as measured by the assessment of individual patient pathways by quality of care indicators and other secondary measurements. The aim is to assess if an improvement that seem pertinent, given the referral deficiencies discussed above, also translate into a measurable care difference for the individual patient.

## Methods

### Study design

This study was designed as a cluster-randomized trial with the GP surgery as the clustering unit. The 14 community GP surgeries in the area served by the medical department at the University Hospital of North Norway Harstad (UNN Harstad) were randomized to an intervention or control group. We chose the cluster-randomized design to avoid potential “contamination” between GPs at the same surgery—as could have occurred with individual GP randomization. Randomisation was done by simple drawing by a person not connected to the research team, stratified by town vs countryside location of surgery.

Patients, hospital doctors, and outcome evaluators were blinded to the intervention status of the patient; participating GPs could not be blinded since they actively used the intervention. Further details about the randomization and study methods are described elsewhere [35].

### Intervention

The intervention consisted of the distribution of referral templates—in electronic and paper form—to be used as reference sheets when initiating a new referral to medical outpatients at UNN Harstad. The GPs could choose whether to use the electronic template directly or use the paper template as a reference, when initiating a new referral. The templates were to be used with the referral of new patients in four separate clinical areas: dyspepsia; suspected colorectal cancer; chest pain; and confirmed or suspected chronic obstructive pulmonary disease (COPD). We developed the referral templates based upon national and international literature in collaboration with local specialists in each medical field. To ensure the appropriateness of the templates, we also obtained assessments from specialists at other Norwegian hospitals. To promote adoption of the intervention, we included only information perceived as imperative in the referrals in the final templates. As an example, the items in the referral template for patients with dyspepsia appear in Table 1; other templates are available on request. The templates were distributed at educational or lunch meetings, and follow-up visits were provided twice a year during the inclusion phase. It was intended that the intervention referrals within the project would be sent to a specific electronic address at UNN Harstad to enable assessment of intervention uptake. The intervention was in use from September 2011 to November 2013 and stopped after the planned period of approximately 2 years [35]. The control group followed normal referral practice.

**Table 1:** Referral template for patients referred with dyspepsia

Item no.	Text item
1	Dysphagia
2	Odynophagia
3	Anorexia
4	Weight loss
5	Haematemesis
6	Melaena
7	Vomiting
8	Medications (especially NSAID^a^, acetylsalicylic acid, bisphosphonates)
9	Nocturnal symptoms
10	Symptom duration
11	Previous peptic ulcer disease
12	Previous upper gastrointestinal tract operations
13	Jaundice
14	Cervical lymphadenopathy
15	Hepatomegaly
16	Anaemia
17	If <50 years, *Helicobacter pylori* status

^a^ *NSAID* non-steroidal anti-inflammatory drugs

### Participants

We included all 14 GP surgeries in the geographical area served by UNN Harstad in the randomization process. In 2013, they had a total list size of 39,253 patients. Individual consecutive patients referred from these GP surgeries received study information and a consent form together with their appointment letter from the hospital. They received an oral reminder regarding study participation at their first hospital appointment. Children (<18 years of age) and patients with reduced capacity to consent were excluded from the project. Patients were recruited from September 2011 until February 2014. Further details about the randomization and recruitment processes are described elsewhere [35].

### Sample size

In the actual study, the intraclass correlation coefficient (ICC) turned out to be 0.02 (95% CI, 0.00–0.06). Estimating the sample size based on the study effect estimates and this ICC with the assumption of 80% power to detect a 10% difference with a *p* value set at 0.05 leads to a total sample size of 94 (84, 124). To detect a 5% difference, a total sample size of 576 (324, unattainable with only 14 clusters) would seem appropriate.

### Data

We retrieved data by manual review of the electronic health records. Electronic retrieval was considered, but seen as too complex and imprecise for clinical quality indicators—a conclusion that has also been made by others [36].

### Outcomes

The present study aimed to assess the quality of the care pathway by the following outcomes measures as outlined in our previous paper [35] and further detailed below.

- Quality indicator score
- Specialist’s subjective quality assessment
- Positive predictive value of referral
- Adequacy of prioritization

#### Quality indicator score

Reviewing the literature few relevant quality indicators assessing information from individual patient’s pathways were found. The quality indicators used to assess quality of care were therefore developed from previous quality-assessment tools and treatment guidelines [13, 16, 18, 37–68]. The indicators were mainly process indicators. Some adaptation was made to align the criteria with locally accepted practice, which has been demonstrated elsewhere when transferring quality criteria to a new context [69]. The indicators were assessed by specialists in the appropriate field and reviewed based on the advice received. However, no formal approach was employed in developing the indicators. The full indicators are available on request, and the set for dyspepsia is available in a translated version as Additional file 1.

Each patient care pathway was scored according to the criteria. The indicator set for each clinical area consisted of a general section and disease specific subsections depending on the final diagnosis in the treatment pathway. Scoring was undertaken by a panel of specialists from different Norwegian hospitals—all blinded to the intervention status of the patient. Eight gastroenterologists, two cardiologists, and two pulmonologists participated. All scorers were independent from the GP surgeries and the hospital involved in the study. To allow assessment of scoring agreement, a subsample of the cases was evaluated by two scorers independently.

The quality indicator score was calculated as an adherence score (number of criteria met divided by number of applicable criteria), as developed by Ashton et al. [70]. If insufficient information was available to ascertain whether or not an applicable criterion was met, it was classed as “not met” [71], thereby producing a conservative quality score. We considered weighting of the criteria based on clinical importance, but this often adds complexity to the analysis without providing insight into the clinical analysis [72]. The total score was calibrated as a percentage to enable comparison and statistical analysis.

#### Subjective quality assessment

The panel of specialists also subjectively scored the treatment pathway for each patient in two ways. Firstly, they provided a quality rating of the treatment process on an ordinal scale of 1–10. Then, they assessed whether the treatment pathway was appropriate with a yes/no response.

#### Positive predictive value of referral

Based on the method of Bennett et al. [73], we calculated the positive predictive value (PPV) of a referral. This represents the chance of a referral leading to a relevant diagnostic or management decision. Adapting this concept from otolaryngology to a medical department, we defined the PPV as the number of referrals that resulted in a histological diagnosis, diagnostic clarification, or change in medical management.

#### Adequacy of prioritization

Prior to including the patients, potential outcome diagnoses within the four clinical areas were grouped into four categories according to severity. As no prior classification was found this was done by the main author based on WHO International Statistical Classification of Disease and Related Health problems 10th revision (ICD-10) disease codes. The groupings were adjusted after feedback from specialists within each clinical field. Each patient was placed in a severity group based on the final ICD-10 code from the hospital medical records. If several codes were set for an individual patient the code belonging to the most severe group was utilized. As an example a final diagnosis of C18.2 (cancer in the ascending colon) would be placed in the most severe group and a final pure symptomatic diagnosis of R19.4 (change in bowel habit) would be placed in the least severe group. When a diagnosis was encountered that could not be categorized according to the pre-planned severity grouping, consensus was achieved among the study organizers before putting it into the appropriate category. This severity grouping was then used to compare the adequacy of the waiting time between the intervention and control groups. Waiting time was defined as the time from the referral was received at the hospital until the first out-patient appointment, measured in days.

### Statistical methods

To assess scoring agreement for the main outcome, we estimated repeatability coefficients [74]. We provide plots of the mean for each pair of scores vs the difference in score between the two raters for the clinical areas of chest pain and COPD (Bland-Altman plots). We did not produce such plots for gastroenterological clinical areas; as it was impossible to define primary and secondary raters for the individual observational pairs when eight raters overlapped, and Bland-Altman plots depend on the sign of the difference between raters.

The cluster design of the present study demanded an analysis that took into account the clustered nature of the data [75]. In this study, we used multi-level regression modelling to evaluate the effect of the intervention on the main outcome (quality indicator score). We employed likelihood ratio tests to assess the appropriateness of the model. To determine the effect of confounders to level one of the model, a change in the regression coefficient for the intervention effect of >10% was considered relevant. Based on prior assessment and subject knowledge, we included patient gender, age, speciality status of hospital doctor, and severity of final diagnosis in the model. We checked effect modification for relevant variables using *p* < 0.05 as the significance level. The CONSORT guideline for cluster randomised trials was adhered to [75].

For the subjective quality assessment, data are presented as medians with interquartile ranges since the values were not normally distributed. In addition, we employed multi-level ordinal regression analysis to confirm the findings. To assess PPV, we used a simple comparison of percentages—without correction for clustering.

We conducted the analyses throughout on an intention-to-treat basis. With this analysis, patients referred from intervention centres were regarded as belonging to the intervention group—even if it was evident that the intervention had not been used by the referring GP for that particular patient. In all analyses the patient was the unit of analysis and a two-level data structure was used.

### Missing data

A small amount of data was missing from the outcome scoring, representing 2/500 (0.4%) for the subjective quality assessment score and 5/500 (1%) for the binary-outcome of adequate treatment process. To allow for a complete data-set analysis, these data were estimated. For the subjective quality score, the two missing values were set as the median value. For the binary outcome, the response was set to yes (numerical value 1) for subjective score values above six and no (numerical value 0) for scores of five and under. Where both the subjective and binary scores were missing, the median value was used for the binary score (yes, numerical value 1).

## Results

### Baseline characteristics

In all, 500 patients were available for analysis in this study: 281 in the intervention arm and 219 in the control arm after exclusion of nine in the intervention and eight in the control arm [34]. No clusters were lost to follow up. There were no significant baseline differences between the patients in the intervention and control arm, as seen in Table 2. The majority of referrals were within the dyspepsia and suspected colorectal cancer clinical areas. More of the GPs in the intervention than in the control group were board certified GPs, but the years of experience were similar in both groups. Significantly more referrals in the intervention arm were sent by female GPs, which probably relates to the higher number of female GPs in the intervention than in the control arm. Most referrals were electronic, but six paper referrals (2.7%) were received in the control arm versus none in the intervention arm. Half (49.5%) of the referrals in the intervention arm were sent to the designated electronic address established for the project; the rest were sent to the standard hospital electronic address.

**Table 2:** Selected baseline characteristics for patients and general practitioner surgeries by intervention status^a^

	Intervention group	Control group	*p* value
Patient demographics ^b^			
Female/male, n (%)	166 (59.1)/115 (40.9)	127 (58.0)/92 (42.0)	0.807
Age, years	59.2 ± 13.6	57.1 ± 15.3	0.101
Urban/rural, n (%)	169 (60.1)/112 (39.9)	121 (55.3)/98 (44.7)	0.272
Clinical group, n (%)			
- Dyspepsia	144 (51.3)	120 (54.8)	
- Suspected colonic malignancy	87 (31.0)	68 (31.1)	
- Chest pain	46 (16.4)	27 (12.3)	
- COPD^c^	4 (1.4)	4 (1.8)	
Hospital appointment with senior house officer/specialist, n (%)	130 (46.3)/151 (53.7)	96 (43.8)/123 (56.2)	0.588
Given right to health care after assessment of referral, yes/no, n (%) ^d^	222 (79.0)/59 (21.0)	168 (76.7)/51 (23.3)	0.587
GP surgery variables^b^			
List size	830.8 ± 208.8	865.5 ± 100.7	0.475
Female/male GP, n (%)	14 (58.3)/10 (41.7)	10 (43.5)/13 (56.5)	0.308
Board certified, yes/no, n (%)	18 (75.0)/6 (25.0)	11 (47.8)/12 (52.2)	0.055
Years experience	16.0 ± 10.4	15.2 ± 11.2	0.784
Number of GPs in surgery	4.3 ± 1.6	4.0 ± 1.6	0.536
- Median	5	5	
- Mode	5	5	
GP referral variables per referral in data set^b^			
Female/male referring GP, n (%)	182 (64.8)/99 (35.2)	93 (42.5)/126 (57.5)	<0.00001
Number of GPs in surgery	4.4 ± 1.5	4.0 ± 1.6	0.003
Specialist, yes/no n (%)	189 (67.3)/92 (32.7)	114 (52.1)/105 (47.9)	0.000556
Years experience	16.2 ± 12.0	15.4 ± 11.7	0.456
Other variables per referral in data set^b^			
Electronic/paper referral, n (%)	281 (100)/0 (0)	213 (97.3)/6 (2.7)	0.005

^a^Two GPs shared two lists at two separate surgeries, both in the intervention group. Weighted analysis that took this into account did not lead to significant changes in the baseline characteristics

^b^Data are presented as mean ± SD or number (%)

^c^ *COPD* chronic obstructive pulmonary disease

^d^After assessment of the referral Norwegian hospital doctors decided whether or not a patient had a legal “right to health care” within a given time

### Scorer agreement

**Fig. 1:**
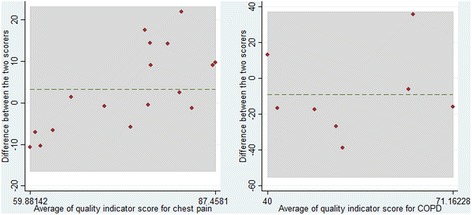
Bland-Altman plot for quality indicator score for area chest pain and chronic obstructive pulmonary disease (COPD)

A subsample of 86 care pathways was scored by two separate specialists to determine concordance between the scorers. For the quality indicator score, the mean difference between the two scoring measurements did not significantly differ from 0, and estimation of the repeatability coefficients, as suggested by Bland and Altman, is presented in Table 3. These suggest a wide variation in scoring between the different scorers. Bland-Altman plots are presented in Fig. 1 for chest pain and COPD since there were only two scorers. It is evident that for chest pain, one of the scorers gave a much higher range of scores than the other. In addition, there is quite clearly a wide variation in scoring between the two scorers for both clinical areas.

**Table 3:** Repeatability coefficient overall and for the four clinical areas

Area	Repeatability Coefficient
Overall	+/− 35.18
Dyspepsia (*n* = 44)	+/− 40.71
Colorectal (*n* = 17)	+/− 23.20
Chest pain (*n* = 17)	+/− 20.25
COPD (*n* = 8)	+/− 46.68

Using absolute values, the mean difference between the scorers was 14% (95% confidence interval [CI], 11.6–16.4) with a coefficient of variation of 80.6%.

For the subjective quality scoring, the repeatability coefficients were also high; Bland-Altman plots for the chest pain and COPD clinical areas showed similar results, with wide variation in scoring (data not shown).

### Quality indicator score

Average quality score, not adjusted for clustering, in the intervention arm was 64.4% (95% CI, 62.4–66.3) and in the control arm 60.0% (95% CI, 57.9–62.2); the averages for each clinical area are presented in Table 4. Using a baseline multi-level model with patients from all clinical areas combined, the ICC was estimated at 0.02 (95%, CI 0.00–0.06). Adding a slope for the intervention status increased the −2 log likelihood of the model and did not make a large change in the residual variance. It was therefore not retained in the model. Postulating a three level data structure by allowing the results to vary randomly at the level of the referring GP only marginally reduced the −2 log likelihood and residual variance of the model. The two level structure proposed in the methods paper was therefore kept. No significant interaction was found. A significant effect of the intervention was seen in the baseline model; however, after correction for relevant confounders, the intervention effect was reduced to 1.80% (95% CI, −1.46 to 5.06, *p* = 0.280). Further regression coefficients appear in Table 5. No clear violation of normality assumptions was noted. Additional modelling for each individual rater revealed no significance of the intervention for any rater (data not shown). Given the significant difference (not corrected for clustering) shown for the dyspepsia group in Table 4 modelling was also performed for each of the four diagnostic groups. No significant effect of the intervention was seen, after correction for confounding factors (data not shown).

**Table 4:** Average quality score per diagnostic group, not corrected for clustering^a^

	Intervention	Control	*p* value
Dyspepsia	62.0 (59.2–64.8)	57.2 (54.1–60.3)	0.023
Suspected colorectal malignancy	65.0 (61.5–68.3)	61.4 (58.3–64.5)	0.138
COPD	48.3 (11.9–84.7)	51.0 (29.0–73.0)	0.847
Chest pain	72.1 (68.5–75.7)	70.8 (65.2–76.4)	0.669

^a^Presented as mean and 95% confidence interval

**Table 5:** Effect estimates for intervention on quality score

	Regression coefficient	95% CI	*p* value
Crude^a^	4.33	1.39–7.27	0.004
Adjusted^b^	1.80	−1.46–5.06	0.280
- Patient gender (male)	1.43	−1.45–4.32	0.330
- Patient age (centred)	0.05	−0.05–0.15	0.314
- Doctor in training^c^ vs. specialist	−5.40	−8.21 to −2.60	<0.001
- Severity of final diagnosis			0.001^*^
- Not severe^d^			
- Less severe	3.20	0.11–6.29	
- Severe	6.20	1.70–10.69	
- Very severe	8.44	−0.17 to 17.05	
- Quality of referral	0.09	0.03–0.15	0.004

^a^Baseline model with intervention effect and random intercept

^b^Adjusted for variables listed in table

^c^Doctor in training (resident) reference

^d^Reference

^*^ *p* value for trend

### Subjective quality score

**Fig. 2:**
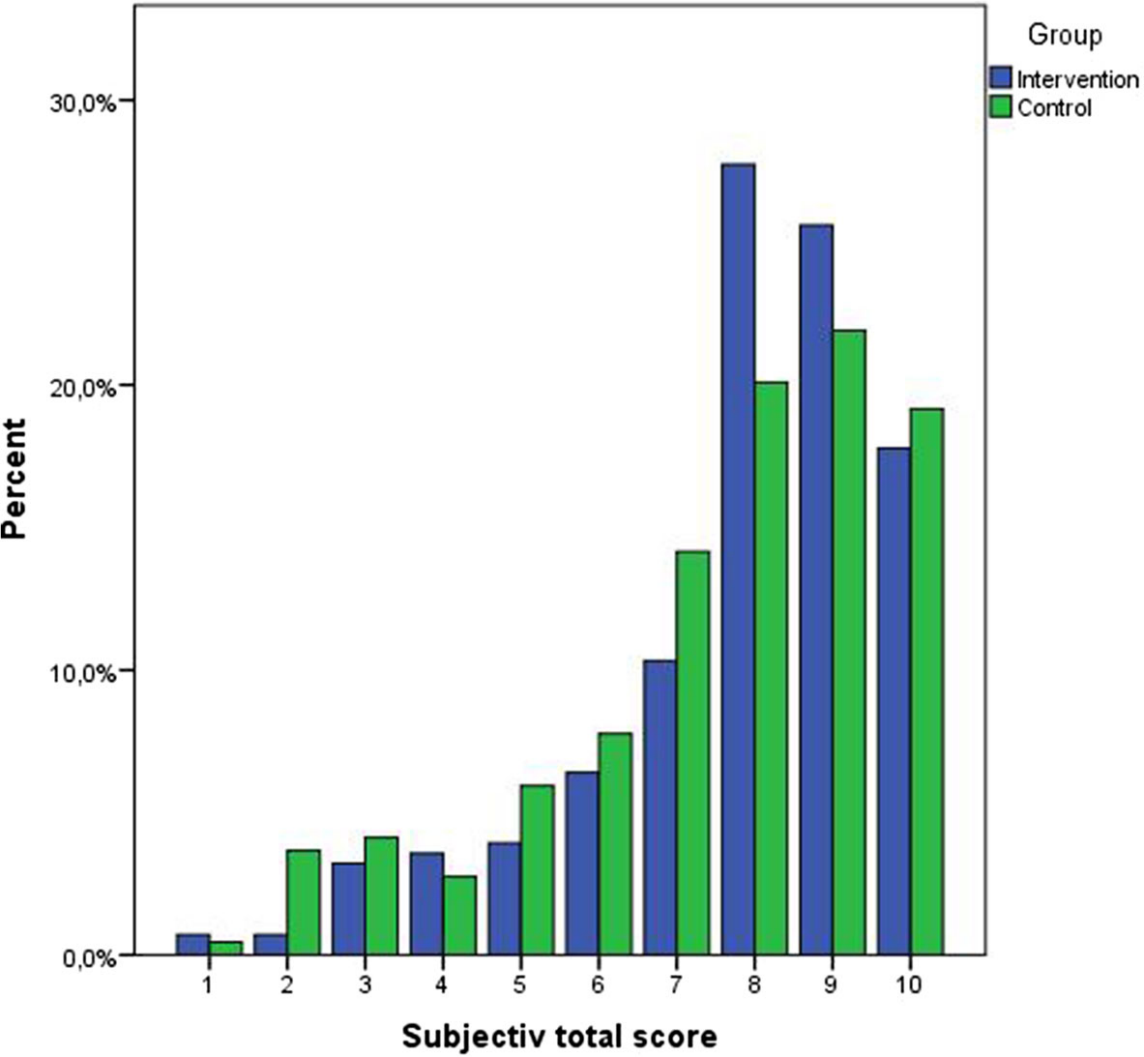
Subjective quality score (1–10) for intervention and control groups

The subjective quality rating was done on an ordinal scale of 1–10. As evident in Fig. 2, the variable was not normally distributed. Overall, the median in the intervention arm and control arm was 8, with an interquartile range of 2. Table 6 presents the median and interquartile range by clinical area and intervention status. No difference between the intervention and control arms appeared in the graph or interquartile ranges. This was confirmed with a multi-level ordinal regression model, in which no difference was noted (data not shown). No difference was observed between the intervention and control arms in the binary(yes/no) assessment of patient pathway appropriateness (data not shown).

**Table 6:** Median of subjective total score^a^

	Intervention	Control
Dyspepsia	8 (2)	7.5 (3)
Suspected colorectal malignancy	9 (2)	9 (2)
COPD	4 (7)	4.5 (5)
Chest pain	8 (2)	8 (2)

^a^Presented as median and interquartile range

### PPV of referral

Table 7 shows the number of patients who had a histological diagnosis, diagnostic clarification, or change in medical management as a result of their outpatient appointment. There were some missing items: this was because part of the scoring sheet with the PPV scoring box appeared on a separate page and may therefore have been overlooked. No clear difference was evident between the intervention and control arms.

**Table 7:** Tabulation of positive predictive value (PPV) of referral, not corrected for clustering^a, b^

	Intervention	Control
Histological diagnosis^c^		
- Yes	86 (37.2)	67 (35.6)
- No	137 (59.3)	112 (59.6)
- Missing	8 (3.5)	9 (4.8)
Diagnostic clarification		
- Yes	220 (78.3)	164 (74.9)
- No	51 (18.2)	46 (21.0)
- Missing	10 (3.5)	9 (4.1)
Change in medical management		
- Yes	154 (54.8)	105 (48.0)
- No	117 (41.6)	105 (48.0)
- Missing	10 (3.6)	9 (4.1)
PPV total		
- Yes	243 (86.5)	183 (83.6)
- No	28 (10.0)	27 (12.3)
- Missing	10 (3.5)	9 (4.1)

^a^Numbers are presented as positive outcomes in absolute numbers and percentages

^b^No significant differences seen between intervention and control groups

^c^Only for the clinical areas of dyspepsia and suspected CRC (not relevant for COPD and chest pain), *n* = 419

### Adequacy of prioritization

The average waiting time (time from referral to first outpatient appointment) was 46 days (95% CI, 42–50) in the intervention arm and 49 days (95% CI, 43–55) in the control arm (*p* = 0.364, *t* test). The waiting times for the four separate clinical areas appear in Table 8, with no significant differences between the intervention and control arms. The large difference in the COPD area is due to small numbers (*N* = 8) and random difference. The average waiting time stratified by intervention or control status and severity of final diagnosis is presented in Table 9. No significant differences were observed between the intervention and control arms. In addition, no definite trend was seen in the waiting time across severity groups—except that the waiting time was significantly shorter for patients with a final diagnosis classed as “very severe” than with the three other severity groupings (*p* = 0.01, *t* test). These average values are not corrected for clustering; however, a simple multi-level model with waiting time as the outcome variable and intervention status as predictor suggested very little effect of clustering, with an estimated ICC of <0.00001. In addition, allowing for clustering in the estimation of the mean led to narrower CIs, which is counterintuitive.

**Table 8:** Average waiting time by clinical area^a^

Clinical area	Intervention group	Control group
Dyspepsia	41 (28.6)	47 (46.3)
Suspicion of CRC	40 (31.4)	43 (37.5)
Chest pain	69 (42.5)	69 (39.1)
COPD	108 (59.6)	78 (44.3)

^a^ Numbers are days (rounded to whole days) with SD in brackets; no significant differences between intervention and control groups

**Table 9:** Average waiting time by severity of final diagnosis ^a^

Severity of final diagnosis	Intervention group	Control group
Not severe (*n* = 190)	47 (35.4)	53 (40.6)
Less severe (*n* = 227)	45 (32.3)	49 (49.1)
Severe (*n* = 68)	55 (42.8)	41 (31.8)
Very severe (*n* = 15)	22 (14.0)	26 (17.7)

^a^Numbers are days (rounded to whole days) with SD brackets; no significant differences between intervention and control groups

Waiting time was not normally distributed. To assess further the effect of the intervention on prioritization, we divided waiting times into deciles and used ordinal logistic regression, with waiting times in deciles as the dependent variable and severity group as predictor. We conducted a separate analysis for the intervention and control arms. This suggested a significant trend in the control arm only, as shown in Table 10. However, the significant effect found in the control arm did not persist if the variable waiting time was divided into ten groups with set intervals (41, 82, 123 … 410) rather than deciles. By way of sensitivity analysis, we also checked the analysis using a multi-level model; however, this did not represent the data significantly better, and so for simplicity we retained the one-level model. Also, standard linear regression did not show any significant variation in waiting time based on the severity score (data not shown); this, though, should be interpreted with caution since the variable was not normally distributed.

**Table 10:** Ordinal regression of waiting time (in deciles) versus severity of final diagnosis

Severity of final diagnosis	Regression coefficient intervention group ^*^	Regression coefficient control group ^**^
Not severe ^a^		
Less severe	−0.77 (−0.53 to 0.38)	−0.33 (−0.84 to 0.17)
Severe	.41 (−0.24 to 1.06)	−0.59 (−1.32 to 0.14)
Very severe	−1.42 (−2.41 to −0.42)	−1.90 (−4.06 to 0.27)

^a^Reference category

^*^ *p* = 0.333 for trend

^**^ *p* = 0.032 for trend

## Discussion

In the present study, we aimed to assess whether implementing a referral intervention would lead to improved quality of care for medical outpatients. We have previously shown that the referral quality did increase [34], however there was no clear effect on the quality indicator score, subjective quality score, or PPV of referrals, as detailed above. In addition, there was no evidence that improving referrals enhanced prioritization at the hospital; in one analysis, prioritization even seemed more precise in the control arm. Hence, it would appear that the use of referral templates did not generate a clear clinical benefit for the individual patients.

In addition to the study limitations discussed below, several factors may explain the lack of effect. First, it is possible that care for patients has improved but that the measurement instruments and outcomes have been unable to quantify it. Guidelines and clinical practice allow some flexibility for the treating clinician, whereas quality criteria often are rigid [76]. Thus, an ideal patient pathway—as was the goal in this study—will not necessarily be represented by 100% adherence to any given set of quality criteria; there will always be some level of subjectivity in the assessment of quality for each individual patient pathway. In future studies, therefore, even more effort is necessary to develop precise, valid outcomes measures to ensure that any potential effect is documented. The use of a mixed-methods approach may also help identify improvements that are hard to quantify; such an approach is regarded as especially useful in health services research [77]. Second, it is possible that the referrals in the control arm were of sufficiently high quality to ensure adequate referral assessment and prioritization at baseline. As such, the scope for improvement was small and therefore difficult to measure. As such further studies in areas with more varied referral quality may allow the effects of referral interventions to be quantified more precisely. Third, referrals are only part of the complex care pathway, and it is possible that improvement of only one part is insufficient to result in quantifiable improvements of the entire process. Other factors—medical, organizational, and individual—may also govern the process.

We have been unable to locate many comparable studies that aimed to assess the effect of a referral intervention on the further patient pathway in hospital—a shortcoming also addressed in a Cochrane review on referral intervention [31]. There are, however, some exceptions, with limited findings that are in line with those of the current project. In a UK study a referral intervention led to improved referral content, but it did not increase the amount of organic pathology revealed among those referred for colonoscopy. The authors commented that the value of the intervention may have been reduced by limited uptake [78]. In the study by Bennett et al. noted above, more appropriate patients were referred, but no information was presented regarding hospital management [73]. In a urology study, the implementation of education meetings and referral guidance led to a reduction in waiting time and an increase in the probability of receiving a management decision at the first appointment, but no difference in patient outcomes was found [79]. In Norwegian mental health services, a study is underway attempting to explore the effect of the quality of referrals on patient and organizational outcomes [80].

One way of promoting the use of referral interventions would be to make them a mandatory part of the referral process: they could appear as drop-down menus together with the relevant clinical information. This procedure would remove problems with uptake of the intervention and enable a more precise determination of the intervention effects. However, the present study found no clear effect of the referral templates, and, as seen in Tables 9 and 10, the prioritization was equally good in the control arm. It therefore seems that there are factors other than the pure informational quality in the referrals that guide the hospital clinician in identifying the most ill patients. It is possible that more subtle clinical details would disappear if the ability to enter free text were completely removed. Hence, the full implementation of obligatory referral guidance should occur only after further assessment has shown it to be of clinical importance.

This study found no significant effect of the intervention. We included at total of 500 patients, with 281 in the intervention and 219 in the control arm. Given the sample size indicated above this means that the study was well powered to detect the 10% change in the quality indicator score that was set as clinically interesting; hence, the risk of a type II error is low. The power calculations do, however, underline the need to increase cluster numbers, rather than cluster size, to increase the power of cluster-randomized studies [81]. The current study would have been underpowered if the ICC had been at the upper end of the confidence interval of the ICC, regardless of how many patients were recruited.

### Strengths and limitations

Certain aspects regarding recruitment and use of the intervention may have hampered the results. The aim of the study was to investigate the use of referral templates in actual clinical practice. In this real-world scenario, it would be pertinent to determine how many of the potential participants were actually recruited. Exact information about this would have required manual searches of outpatient lists and relevant electronic journals—this was incompatible with the ethical approval for the project and current legal regulations. However, indirect evidence indicates that 60% of potential patient participants were recruited. We have no indication that this figure varied between the intervention and control arms. Although we have no indication that the current sample differs from those not recruited the study did not assess this formally due to the constraints mentioned above. In addition, it is not clear how often the referring GPs actively utilized the intervention when initiating new referrals. The designated electronic project address was used approximately 50% of the time, which suggests a fairly modest uptake, although higher than in other studies [78]. This is likely to have attenuated the intervention effect since intention-to-treat analysis was employed. In total, these aspects are unlikely to have led to a significant selection bias, but may have attenuated the intervention effect.

The high variation in scoring among the scorers limits the applicability of the statistical analysis. This study opted to use numerous assessors, instead of just a few, to achieve a manageable assessment workload. The result was that a wide variety of scorers from different hospitals and clinical cultures took part. To try and ensure the validity of the conclusions, we performed subanalyses and ran the models individually for each rater. This of course yielded higher CIs, but the overall effects retained the same sign and magnitude. This was not suprising as the raters were given a mix of control and intervention patient pathways for scoring. We therefore feel that although the variation may limit the generalizability of the measurement instruments, it does not necessarily invalidate the conclusions of this study.

Since health-care quality is not a defined physical entity or even a clearly defined concept, it will always be difficult to measure precisely. Many authors have tried to measure quality of care and hospital quality and have used various ways, even Facebook [82]. The development of quality criteria is often challenging and should be based on accepted standards of care using sound evidence [83]. What is being measured should also represent an important aspect of care for the particular condition. In addition, an indicator has to be clearly defined, and the information must be available [83]. Most criteria in use today are accountability measures, designed to measure adherence to specific actions and employed for accreditation or reimbursement [24, 84]. In the present study, process indicators were developed for the care of patient groups, who ended up with a plethora of diagnoses instead of clearly defined diagnostic groups with simple measurements. This approach is clearly in line with the aim of this study, which was to investigate the use of referral guidance in normal clinical practice. Accordingly, it may be seen as reflecting a strength of the study. However, it added complexity to the development of the study outcome criteria. The criteria employed in this study do not, therefore, fulfil all requirements of ideal process criteria; overall, however, they represent an attempt to quantify the quality in everyday clinical practice at the level of the individual patient. This limits comparability with other studies, but we believe that this approach was more likely to identify the effects of referral intervention since such effects were expected to be subtle rather than obvious.

Another potential limitation is the quality of the source of clinical information. Hospital records were used to obtain the relevant information. Implicitly, this study did not therefore measure if a certain action was performed, but whether the action was performed and documented. Whereas the prospective collection of information from electronic health records is the most thorough way of acquiring information [85], the quality of medical records has been debated for some time [86–88]. Electronic health records have facilitated documentation, but the quality and completeness of the data is still under debate [89]. However, information gathering and assessment were performed the same way for both the intervention and control arms, and there was no indication that the manner of documentation gathering led to information bias.

The main strength of the present study is closely related to its weaknesses. This study was performed in a normal clinical setting without major intervention at any level other than the referral. This real-life approach should ensure that the results are applicable for many other health-care settings where referral from the GP to the hospital specialist is the norm.

## Conclusions

This cluster-randomized trial was designed to assess the impact of a referral intervention on the quality of care and hospital management of patients. No measurable effect on quality of care or prioritization of patients was found. The results were hindered by a limited uptake of the intervention at GP surgeries and inconsistencies in outcome assessment. It seems reasonable to assume that more information in the referral will improve further management, but more stringent assessment may, in future research, be necessary.

## Additional file

Additional file 1: Indicator set for dyspepsia translated into English. (DOC 108 kb)

## Abbreviations

CI: Confidence interval
COPD: Chronic obstructive pulmonary disease
GP: General practitioner
ICC: Intraclass correlation coefficient
ICD-10: International Statistical Classification of Diseases and Related Health Problems 10th Revisions
PPV: Positive predictive value
REK NORD: Regional Ethical Committee for Medical Research in North Norway
UCLA: University of California
UNN Harstad: University Hospital of North Norway Harstad
