# Supplementary material for: The effect of referral templates on out-patient quality of care in a hospital setting: a cluster randomized controlled trial
Source: BMC Health Serv Res. 2017 Mar 7;17:177. doi: 10.1186/s12913-017-2127-1 (PMC5341470; doi:10.1186/s12913-017-2127-1)
Supplement: Additional file 1: — Indicator set for dyspepsia translated into English. (DOC 108 kb) [file 12913_2017_2127_MOESM1_ESM.doc]

**Patient nr:**

***Practical health co-operation – a cluster randomized controlled trial***

**Quality indicators for patients referred with dyspepsia [1-11]**

For questions or further information:

Henrik Wåhlberg

93 80 42 40

77 01 52 59

henrik.wahlberg2@unn.no

**Please fill out the following:**

- Part I
  - 1.1 – *”Work-up”:* To be filled out for every patient
  - 1.2 – *”Common elements”* in treatment: To be filled out for every patient
  - 1.3 – 1.8 – From *”Treatment of gastrointestinal reflux”* to *”Occult GI bleeding”* each relevant subsection is to be filled out according to the patient’s diagnosis
  - 1.9 – *”Subjective quality measures”*: To be filled out for every patient
- Part II – Fill out for every patient

**Part II – Quality indicator set**

Specify as “Not fulfilled” if the criteria is relevant, but the information cannot be located.

| No | Indicator | Fulfilled | Not fulfilled | | Not relevant | |
| --- | --- | --- | --- | --- | --- | --- |
| 1.1 – Work-up | | | | | | |
| 1 | Diagnosis clear at first visit *or* further investigational plan clearly defined |  |  | |  | |
| 2 | Further follow up planned *or* the responsibility for follow up clearly returned to the general practitioner |  |  | |  | |
| 3 | Patients >50 years of age or younger with alarm symptoms* given access to health services within 6 weeks of referral date |  |  | |  | |
| 4 | Should this patient, based on the referral, be given ’right to health care’ according to the national prioritization guidelines  (fulfilled = yes, not fulfilled = no) |  |  | |  | |
| 5 | Gastroscopy only performed if:   - > 50 years of age - < 50 years of age and H. pylori positive *or* anaemia *or* NSAID use - patients who do not respond to PPI or have had recurrence of symptoms after adequate trial of medications |  |  | |  | |
| 6 | At gastroscopy documented information to patient about procedure and consent obtained |  |  | |  | |
| 7 | Medical history of alarm symptoms documented* |  |  | |  | |
| 8 | Medical history of NSAID use documented |  |  | |  | |
| 1.2 – Common elements in treatment | | | | | | |
| 9 | Lifestyle advice, e.g. healthy eating habits, smoking cessation and weight loss given |  |  | |  | |
| 10 | Advice to avoid trigger factors, such as stress, body position, specific foods given |  |  | |  | |
| 11 | For patients with long term medication use advice given about using the lowest effective dose, intermittent dosing and the use of over the counter medication |  |  | |  | |
| 1.3 – Treatment of gastrointestinal reflux | | | | | | |
| 12 | If typical medical history *or* typical gastroscopy findings, full dose PPI offered for 1-2 months *and* a plan for evaluation of effect documented |  |  | |  | |
| 13 | For symptomatic recurrence the lowest effective dose of medications offered |  |  | |  | |
| 14 | Assessment with oesophageal pH measurement offered if the effect of medical treatment, including double dose PPI, is deemed insufficient |  |  | |  | |
| 15 | Referral to surgery only offered when symptoms are bothersome despite maximal medical management |  |  | |  | |
| 1.4 – Treatment of peptic disease | | | | | | |
| 16 | Test for H. pylori performed |  |  | |  | |
| 17 | If H. pylori positive the patient offered/advised eradication |  |  | |  | |
| 18 | If NSAID associated ulceration PPI for 2 months offered *with* eradication if H. pylori present |  |  | |  | |
| 19 | If gastric ulcerations and positive H. pylori retesting to assess eradication and a control gastroscopy planned |  |  | |  | |
| 20 | Full dose PPI offered to patients who are H. pylori negative and do not use NSAID |  |  | |  | |
| 21 | For patients using NSAID withdrawal recommended *or* if no withdrawal possible dose reduction *or* intermittent dosing recommended |  |  | |  | |
| 22 | For patients with a high risk of ulcer recurrences, prophylactic treatment offered (PPI or H2 blocker) |  |  | |  | |
| 23 | For patient with ulcers that do not heal the presence of non-compliance, malignancy, false negative H. pylori test, unintended NSAID use and uncommon diseases (e.g. Crohn, Zollinger-Ellison) evaluated |  |  | |  | |
| 1.5 – Treatment of endoscopically verified non-ulcer dyspepsia | | | | | | |
| 24 | Test for H. pylori performed |  |  | |  | |
| 25 | If H. pylori positive, the indication for eradication assessed |  |  | |  | |
| 26 | If eradication has been given, follow up testing arranged |  |  | |  | |
| 27 | Patient offered treatment with low dose PPI or H2 blocker for one month |  |  | |  | |
| 28 | If persistent symptoms further treatment with low dose PPI/H2 blocker *or* treatment on a per needed basis offered |  |  | |  | |
| 1.6 – Treatment of Barret’s oesophagus/suspicion of Barret’s oesophagus | | | | | | |
| 29 | Findings classified after the Prague classification (CM) |  |  | |  | |
| 30 | Recommendations for taking biopsies followed *or* reasons for other strategy documented   - no known dysplasia: 1 biopsy from each quadrant pr 2 cm + biopsies from suspect areas - previously dysplasia: 1 biopsy from each quadrant pr 1 cm + biopsies from suspect areas |  |  | |  | |
| 31 | Patients follow up planned   - 3 years if no dysplasia - 6-12 months if low grade dysplasia - 3 months if untreated high grade dysplasia |  |  | |  | |
| 32 | If high grade dysplasia is found further adequate treatment planned |  |  | |  | |
| 33 | If symptoms or endoscopic reflux oesophagitis patient offered PPI for 1-2 months |  |  | |  | |
| 34 | A discussion regarding long term PPI use documented |  |  | |  | |
| 1.7 – Coeliac disease | | | | | | |
| 35 | The diagnosis secured by duodenal biopsy *or* reasons for other approach documented |  |  | |  | |
| 36 | Nutritional status assessed by blood tests (ferritin, vitamin B12, folate) |  |  | |  | |
| 37 | Advice given about a gluten free diet and referral to dietary advice provided |  | |  | |  |
| 38 | Information about patient organisation given (Norwegian Coeliac Association) |  | |  | |  |
| 39 | Plan for follow up clearly organised |  | |  | |  |
| 1.8 – Occult GI bleeding | | | | | | |
| 40 | The indication for colonoscopy assessed if positive faecal test for occult blood *or* iron deficiency anaemia *and* normal findings at gastroscopy |  | |  | |  |
| 41 | If occult bleeding and no significant findings at gastroscopy *and* colonoscopy referral to capsule endoscopy *or* repeat endoscopy planned |  | |  | |  |
| 42 | Small bowel biopsies to assess for coeliac disease performed |  | |  | |  |
| 1.9 – Subjective quality assessment | | | | | | |
| 43 | Did this patient pathway in total represent an adequate treatment process (from referral to end of treatment process)  (fulfilled = yes, not fulfilled = no) |  | |  | |  |
| 44 | Give a total score for the treatment pathway on a scale of 1-10  (1: inadequate treatment process – 10: excellent treatment process) | 1 2 3 4 5 6 7 8 9 10 | | | | |

* Alarm symptoms

- weight loss
- progressive dysphagia
- odynophagia
- repeated vomiting
- signs of GI bleeding
- family history of upper GI tumour
- jaundice
- palpable mass
- previous medical history of ulcers
- >50 years old with new symptoms
- previous oesophageal or gastric cancer

**Part II - ”Positive predictive value” of referral [12]**

Has the health care delivered lead to;

|  | Yes | No |
| --- | --- | --- |
| a) a histological diagnosis |  |  |
| b) a diagnostic clarification |  |  |
| c) a change in medical management |  |  |

Reference List

1. *Dyspepsia: Managing Dyspepsia in Adults in Primary Care.* Newcastle upon Tyne; 2004.

2. **American Gastroenterological Association Medical Position Statement: Evaluation of Dyspepsia.** *Gastroenterology* 2005, **129:**1753-1756.

3. Talley NJ, Vakil N: **Guidelines for the management of dyspepsia.** *Am J Gastroenterol* 2005, **100:**2324-2337.

4. **Riktlinjer handläggning av gastroesofageal refluxsjukdom.** 2008. Svensk Gastroenterologisk Förening.

5. Valle PC, Breckan RK, Amin A, Kristiansen MG, Husebye E, Nordgard K, Mortensen L, Kildahl-Andersen OA, Wessel-Berg AM: **"Test, score and scope": a selection strategy for safe reduction of upper gastrointestinal endoscopies in young dyspeptic patients referred from primary care.** *Scand J Gastroenterol* 2006, **41:**161-169.

6. Longstreth GF: **Approach to the patient with dyspepsia.** In *UpToDate*. Edited by Edited by Basow DS. Waltham, MA: 2009.

7. **American Gastroenterological Association Medical Position Statement on the Management of Barrett's Esophagus.** *Gastroenterology* 2011, **140:**1084-1091.

8. Jones R, Hunt C, Stevens R, Dalrymple J, Driscoll R, Sleet S, Blanchard SJ: **Management of common gastrointestinal disorders: quality criteria based on patients' views and practice guidelines.** *Br J Gen Pract* 2009, **59:**e199-e208.

9. Ciclitira PJ, Dewar DH, McLaughlin SD, Sanders DS: *The Management of Adult with Coeliac Disease.* 2010.

10. Fisher L, Lee Krinsky M, Anderson MA, Appalaneni V, Banerjee S, Ben Menachem T, Cash BD, Decker GA, Fanelli RD, Friis C et al.: **The role of endoscopy in the management of obscure GI bleeding.** *Gastrointestinal Endoscopy* 2010, **72:**471-479.

11. Bull-Henry K, Al Kawas FH: **Evaluation of occult gastrointestinal bleeding.** *Am Fam Physician* 2013, **87:**430-436.

12. Bennett K, Haggard M, Churchill R, Wood S: **Improving referrals for glue ear from primary care: are multiple interventions better than one alone?** *J Health Serv Res Policy* 2001, **6:**139-144.
